# V7 ENB‐guided thoracoscopic sublobectomy for stage IA synchronous multiple primary lung cancer

**DOI:** 10.1111/1759-7714.14706

**Published:** 2022-10-22

**Authors:** Kun Wang, Yu Zhang, Mengchao Xue, Yueyao Wang, Rongyang Li, Libo Si, Weiming Yue, Hui Tian

**Affiliations:** ^1^ Department of Thoracic Surgery Qilu Hospital of Shandong University Jinan China; ^2^ Department of Pathology Qilu Hospital of Shandong University Jinan China

**Keywords:** electromagnetic navigation bronchoscopy, multiple primary lung cancer, sublobectomy, video‐assisted thoracic surgery

## Abstract

**Background:**

An increasing number of patients are being diagnosed with synchronous multiple primary lung cancer (SMPLC) with the popularization of lung cancer screening programs. However, a strategy for accurate location and suitable surgery therapy is still lacking. The present study aimed to explore the accuracy and feasibility of electromagnetic navigation bronchoscopy (ENB)‐guided thoracoscopic sublobectomy for stage IA SMPLC.

**Methods:**

Patients with SMPLC who underwent ENB‐guided sublobectomy from January 2020 to June 2022 were enrolled in this study. The analysis of localization accuracy of ENB and surgical outcome was conducted.

**Results:**

Overall, 138 patients with 353 malignant nodules were enrolled. The tumor size was 0.7 cm (range from 0.5 to 1.1 cm). ENB localization was performed on 162 nodules, and a customized scoring system was developed to evaluate localization accuracy. The success rate of localization was 98.3% (178/181). Notably, localization accuracy was positively correlated with bronchial signs (*p* < 0.01) and negatively correlated with the distance from the nodule to the pleura (*p* = 0.02). All nodules were completely resected. Operation time, drainage volume on the third postoperative day, and catheter time were significantly correlated with the resected lesion numbers (*p* = 0.009, *p* = 0.004, and *p* = 0.01, respectively).

**Conclusions:**

ENB‐guided uniportal video‐assisted thoracoscopic sublobectomy provides accurate preoperative localization and avoids unnecessary lung resection of patients with stage IA SMPLC. However, complete resection of multilocation nodules (more than four lesions) increases the risk of postoperative complications. A new combined treatment strategy for SMPLC should be explored.

## Graphical abstract

(a) The ENB platform system. (b) Reconstruction visual virtual airway routes. (c) Bilateral tracheal path registration was performed using a guiding wire. (d) The indocyanine green was used for fluorescence thoracoscopic technique. (E) ENB‐guided uniportal video‐assisted thoracoscopic sublobectomy is feasible and effective in patients with stage IA SMPLC. This technique provides accurate preoperative localization of multiple pulmonary nodules. ENB: electromagnetic navigation bronchoscopy; SMPLC: synchronous multiple primary lung cancer.

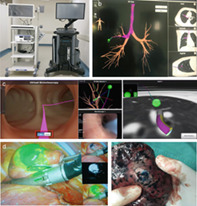

## INTRODUCTION

Recently, with the advent of people's health awareness and the application of high‐resolution computed tomography (CT), the detection rate of multiple primary lung cancer (MPLC) has been increasing.^1^, ^2^ As a unique subtype of lung cancer, the concept and diagnostic criteria of MPLC were first proposed by Martini.^3^ MPLC can be categorized into synchronous multiple primary lung cancer (SMPLC) and metachronous multiple primary lung cancer, according to the occurrence time of primary malignancy. Although the pathological diagnosis strategy of SMPLC has been updated from traditional histological features to molecular classification based on molecular and gene mutation typing,^4^, ^5^, ^6^ the surgical management consensus on SMPLC is still lacking.

Preoperative localization of early‐stage SMPLC is another critical step for minimal and precise invasiveness. SMPLC is characterized by multiple lesions and some small nodules that are difficult to locate or identify by observation and palpation.^7^, ^8^ In addition, electromagnetic navigation bronchoscopy (ENB) is a visual‐assisted localization tool that might reach any position through electromagnetic technology and 3D image reconstruction.^9^, ^10^ Compared with traditional localization methods, such as CT‐guided localization and intraoperative ultrasound‐assisted localization, ENB has the advantages of nonradiation, minimally invasive, and fewer complications.^11^ Currently, the effectiveness and accuracy of ENB guided uniportal video‐assisted thoracoscopic (Uni‐VATS) sublobectomy in treating SMPLC are still unclear. Accurate localization and appropriate surgical resection range based on the number and location of nodules need to be explored to achieve a satisfactory prognosis.

Therefore, this study included all stage IA patients with SMPLC who underwent ENB‐guided Uni‐VATS sublobectomy at Qilu Hospital of Shandong University from January 2020 to June 2022. The accuracy and safety of ENB were evaluated by a self‐made accuracy localization scale, and the feasibility of sublobectomy was analyzed by short‐term perioperative outcomes.

## METHODS

### Patients

A single‐centered retrospective research was conducted to analyze the patients who underwent preoperative ENB‐guided Uni‐VATS sublobectomy at the Department of Thoracic Surgery, Shandong University of Qilu Hospital, between January 2020 and June 2022. The same surgical team performed ENB and Uni‐VATS. A total of 138 patients diagnosed with SMPLC by histological type assessment (immunobiological staining and cytological features analysis) referring to the SMPLC diagnostic criteria were enrolled in this study.^4^, ^12^ The inclusion criteria were as follows: (i) Chest CT showed two or more nodules in the ipsilateral lung field with a diameter<3 cm. (ii) Preoperative chest CT indicated that at least one lesion was difficult to locate or identify by observation and palpation. (iii) Postoperative pathology confirmed that all resected nodules belonged to lung cancer, including carcinoma in situ, microinvasive adenocarcinoma, invasive adenocarcinoma, and other types of primary lung cancer such as squamous cell carcinoma, carcinoid, and lung small cell carcinoma, among others. (iv) No personal history of malignancy. The exclusion criteria were as follows: (i) Patients with an impaired cardiopulmonary function who cannot tolerate surgery. (ii) Patients with pathological results of resected nodules were benign tumors including atypical adenomatous hyperplasia, hamartoma, and tuberculoma, among others. (iii) Distant metastasis or lymph node metastasis was reported in preoperative evaluation.

### ENB localization procedure

**FIGURE 1:**
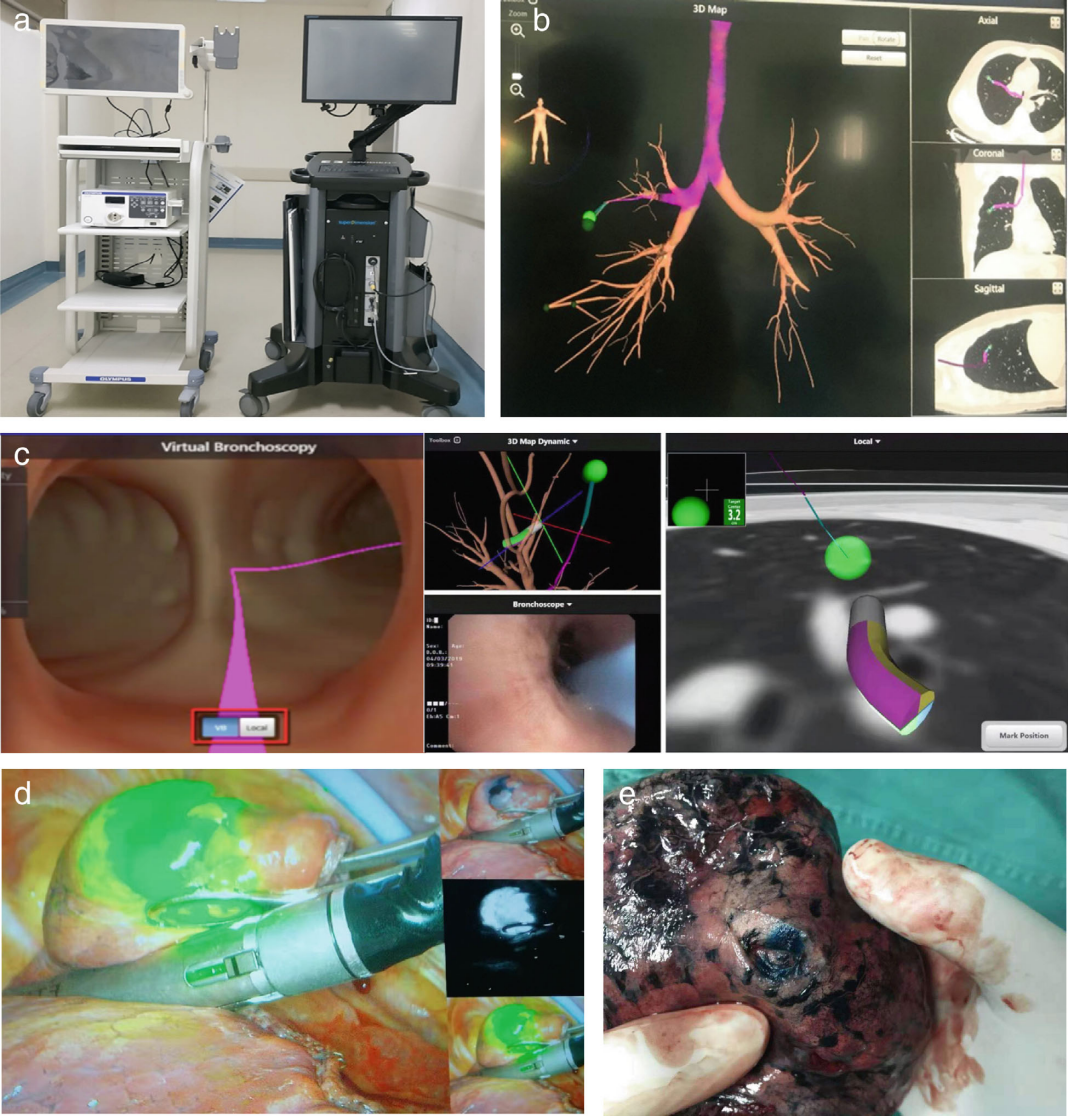
Electromagnetic navigation bronchoscope guided thoracoscopic procedure: (a) V7 electromagnetic navigation machine; (b) reconstructed virtual path to locate the target nodule; (c) electromagnetic navigation screen of the magnetic probe; (d) Indocyanine green fluorescence thoracoscopic technique in single‐port thoracoscopic sublobectomy; (e) the lesions were accurately localized after resection.

Each patient underwent an enhanced chest CT scan preoperatively. Bronchial sign classification on CT was categorized according to the Tokoro classification standard.^13^, ^14^ The classes were as following: Class 0: no bronchial signs; Class 1: lesion was close to small airway sit, Class 2: the airway directly led to the lesion. (i) The ENB platform system (Figure 1a; superDimension Navigation System version 7.0, Medtronic) extracted digital imaging and communications in medical images and reconstructed visual virtual airway routes (Figure 1b). In addition, the distance from the terminal bronchus to the lesions was recorded along the navigation track through established three‐dimensional coordinates. (ii) The patient was under general anesthesia with a laryngeal mask, and bilateral tracheal path registration was performed using a guiding wire (Figure 1c). The procedure was incomplete until subsequent registration of the bilateral main bronchus and trachea through a guide wire. (iii) Under virtual navigation guidance, a guide wire with a sheath was inserted into the target lesion. We injected 0.2 ml indocyanine green and 0.2 ml melan following 2 ml air through the guide sheath to label the deep nodules. Indocyanine green was used for the fluorescence thoracoscopic technique (Figure 1d), and melan‐A was used for dye marker assessment (Figure 1e). The distance from the staining area to the lesion was measured and recorded on the resected specimens. Subsequently, a customized scoring system was developed to score the accuracy of localization. Accuracy scores of 4 and 5 were identified as accurate localization.

### Uniportal video‐assisted thoracoscopic surgery

Localization and thoracoscopic surgery were performed in the same operating theater. When localization was complete, the patient was changed to a lateral decubitus position and the electromagnetic positioning plate behind the patient was removed. Then, the anesthesia one‐lung ventilation mode was maintained using the bronchial blocker technique. Furthermore, the surgical position was changed to the right or left lateral decubitus position according to the requiring side of the patient. Considering the small size of all nodules in these patients, we performed unilateral sublobectomy containing the major lesions and selective lymph node dissection to preserve lung function and speed up patient recovery. Regular follow‐up or elective surgery was recommended for patients with smaller nodules on the other side. Completely thoracoscopic sublobectomy included wedge resection, single segmentectomy, and segmentectomy combined with adjacent subsegmentectomy, single and combined subsegmentectomy.

### Statistical analysis

The mean and standard deviation were utilized to represent the continuous variables subject to normal distribution, and the mean values were compared using a *t*‐test. The non‐normally distributed continuous variables are represented by a median and interquartile range, and Mann–Whitney U test was performed to compare the distributions among subgroups. Categorical variables were displayed as ratios, and variables among different groups were compared using Fisher's exact test. Patients were categorized according to a customized localization accuracy evaluation scale. In addition, ordinal logistic regression analysis was conducted for localization accuracy score analysis. *p*‐values ≤0.05 were considered statistically significant. Statistical analyses were conducted using IBM SPSS statistical package for social sciences 25 software (IBM Corporation).

## RESULTS

### Patient and nodule characteristics

Overall, 138 patients with 353 small nodules who underwent ENB guided single‐port thoracoscopic sublobectomy were enrolled in this study. Patient and lesion characteristics are presented in Tables 1 and 2, respectively. The median age was 57 years (range 49–64). Of these patients, 99 (71.74%) were male, and 21 (15.22%) had smoking history. In addition, 19 (13.77%) patients had a family history of malignancy. Fifty‐four patients had underlying diseases, including 42, eight, and four with cardiovascular disease, diabetes mellitus, and chronic pulmonary disease, respectively.

**TABLE 1:** Basic characteristics of the patients

Characteristic	Counts, n (%)	Median	Range
Age		57	49.00–64.25
Sex			
Male	39 (28.26)		
Female	99 (71.74)		
Smoking history			
Yes	21 (15.22)		
No	117 (84.78)		
Comorbidity			
Family history	19 (13.77)		
Cardiovascular diseases	42 (30.43)		
Diabetes mellitus	8 (5.80)		
Chronic pulmonary disease	4 (2.90)		
Nodule number			
2	85 (61.60)		
3	36 (26.09)		
4	10 (7.25)		
5	7 (5.07)		
Lung function			
FEV1, L		2.48	2.12–2.83
FEV1/FVC ratio		78.19	74.58–78.19
ASA scores		2	2.00–2.00
CEA, ng/ml		1.82	1.24–2.67
CA125, U/ml		8.71	6.30–13.13
NSE, ng/ml		18.75	14.98–24.07
CYFRA21‐1, ng/ml		1.81	1.38–2.50
SCC, ng/ml		0.90	0.70–1.34
proGRP, pg/ml		40.00	33.72–49.25

Abbreviations: ASA, American Society of Anesthesiologists; CA125, carcinoma antigen 125; CEA, carcinoembryonic antigen; CYFRA21‐1, cytokeratin 19 fragments; ENB, electromagnetic navigation bronchoscopy; NSE, neuron‐specific enolase; proGRP, progastrin‐releasing peptide; SCC, squamous cell carcinoma antigen.

**TABLE 2:** Characteristics of nodules resected by VATS sublobectomy

Characteristic	Counts, n (%)	Median	Range
Total number (per‐patient basis)		2.00	2.00–3.00
Nodule size, cm		0.70	0.50–1.10
Radiological appearance			
pGGO	139 (39.38)		
mGGO	164 (46.46)		
Partially solid	34 (9.93)		
Solid	16 (4.53)		
Distance from nodules to visceral pleura, cm		2.47	1.91–3.26
Bronchus sign on CT			
Class 0	50 (14.16)		
Class 1	121 (34.28)		
Class 2	182 (51.56)		
Postoperative pathology			
AIS	116 (32.86)		
MIA	131 (37.11)		
IA	96 (27.20)		
Lepidic adenocarcinoma	30 (31.25)		
Acinar adenocarcinoma	43 (44.79)		
Papillary adenocarcinoma	14 (14.58)		
Micropapillary adenocarcinoma	4 (4.17)		
Solid adenocarcinoma	5 (5.20)		
IMA	5(1.41)		
SCC	2 (0.57)		
LSCC	1 (0.28)		
Carcinoid	2 (0.57)		

Abbreviations: AIS, adenocarcinoma in situ; CT, computed tomography; IA, invasive adenocarcinoma; IMA, invasive mucinous adenocarcinoma; LSCC, lung small cell carcinoma; mGGO, mixed ground glass opacity; MIA, minimally invasive adenocarcinoma; pGGO, pure ground‐glass opacity; SCC, squamous cell carcinoma; VATS, video‐assisted thoracic surgery.

**FIGURE 2:**
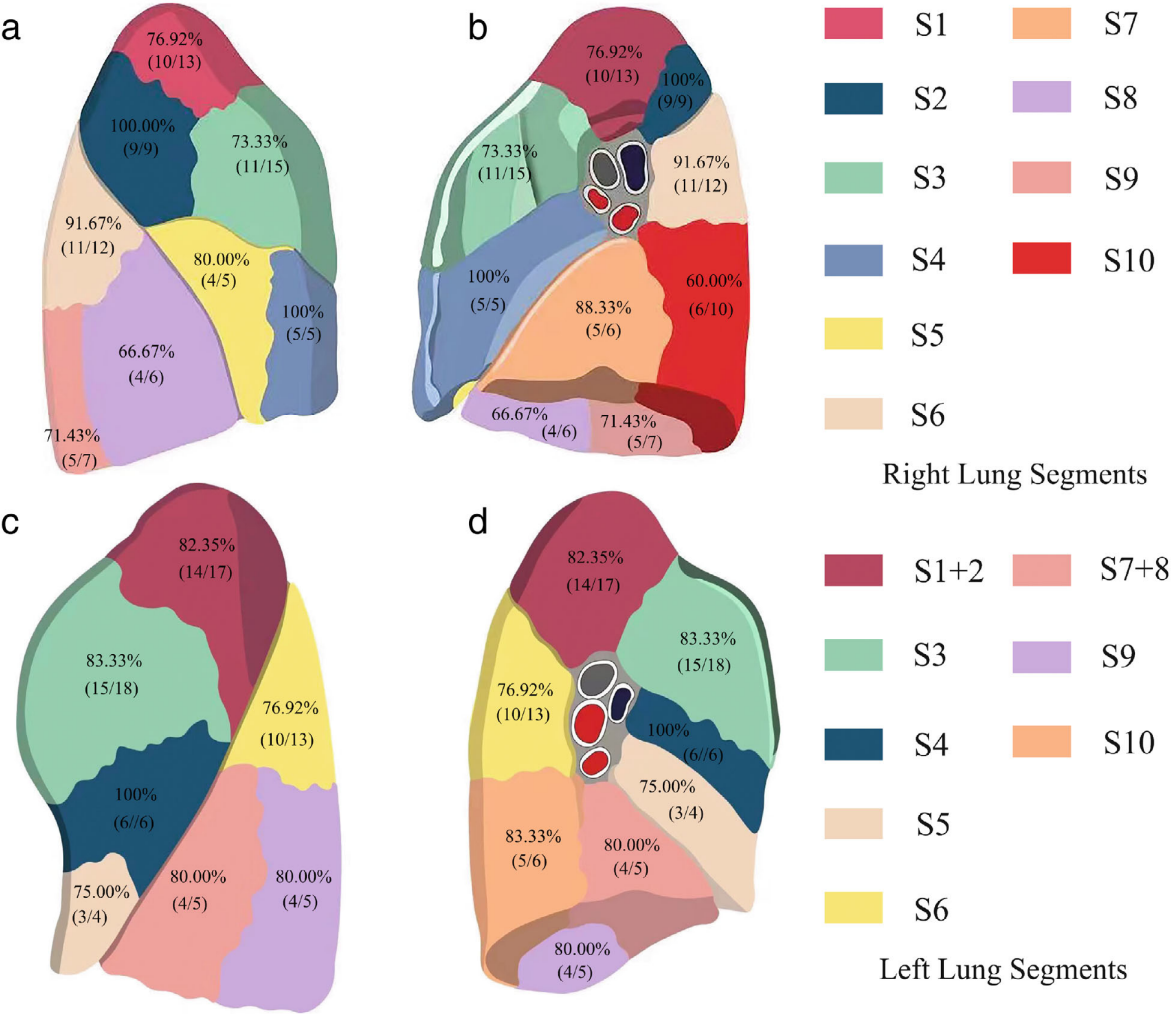
The distribution and localization accuracy rates in different lung segments. (a) Lateral and (b) medial aspects of the right lung segments; (c) lateral and (d) medial (aspects of the left lung segments.

A total of 353 nodules were resected in 138 patients. Furthermore, 85 (61.60%), 36 (26.09%), 10 (7.25%), and seven (5.07%) patients had two, three, four, and five nodules, respectively. The median lesion size was 0.7 cm (range 0.5–1.1 cm). Figure 2 shows the distribution of nodules in each lung segment. Overall, 303 nodules showed bronchial signs, with 182 (51.56%) indicating bronchial reaching in CT reconstructed images (Class 2). Most nodules on CT images were mGGO and pGGO, with 164 (46.46%) and 139 (39.38%), respectively. There were only 34 partial solid nodules and 16 solid nodules in radiological images. The mean distance between the pleura and nodule was 2.47 cm (range 1.91–3.26 mm). Electromagnetic navigation localization was performed on 162 nodules of 138 patients. Among them, 117 (84.78%), 18 (13.04%), and three (2.17%) patients had one, two, and three targets, respectively. Furthermore, 161 (99.83%) nodules were well stained (the dye was not blurring or extravasating) and one nodule did not show dye labeling in the visceral pleura., and 131 were identified as accurately localized. The customized electromagnetic navigation accuracy positioning scoring system is presented in Table 3.

**TABLE 3:** The customized electromagnetic navigation accuracy positioning scoring system

Localization accuracy score	Distance from lesion to dye mark	Nodule numbers, n%
1	No marker	1 (0.62%)
2	Distance > 20 mm	5 (3.10%)
3	10 mm < distance ≤ 20 mm	25 (15.43%)
4	6 mm < distance ≤ 10 mm	81 (50.00%)
5	Distance ≤ 6 mm	50 (30.86%)

### Ordinal logistic regression analysis

To further explore the risk factors of electromagnetic navigation localization accuracy, we performed ordinal logistic regression analysis based on the nodular properties and imaging feature data. The logistic regression model showed a good fit effect through parallel regression assumption (*p* = 0.993). Logit probit was set as link function. As shown in Table 4, the localization accuracy of ENB (estimate = −1.27, *p* < 0.01) will be reduced if the nodules are a long distance from the pleura. Nodules with no bronchial signs were risk factors for low localization accuracy scores (estimate = −1.17, *p* = 0.02). The localization accuracy of ENB was low when the postoperative pathological results were malignant (all *p* < 0.01).

**TABLE 4:** Ordinal logistic regression analysis results in ENB‐guided patients

Parameters	Estimate	95% confidence interval		*p* value
		Lower bound	Upper bound	
Nodule size, cm	−0.63	−2.22	0.83	0.37
Distance from nodules to visceral pleura, cm	−1.27	−1.63	0.92	< 0.01
Nodule location				
RUL	−0.09	−1.11	0.94	0.87
RML	1.39	−0.17	2.95	0.08
RLL	−0.63	−1.65	0.40	0.23
LUL	−0.17	−1.17	0.82	0.73
LLL	0^a^			
Radiological appearance				
mGGO	−2.86	−6.22	0.49	0.09
pGGO	−2.07	−5.45	1.31	0.23
Partially solid	−3.36	−7.15	0.43	0.08
Solid	0^a^			
Bronchus sign on CT				
Class 0	−1.17	−2.15	−0.19	0.02
Class 1	−0.65	−1.40	0.09	0.09
Class 2	0^a^			
Postoperative pathology				
AIS	−21.26	−25.38	−17.13	< 0.01
MIA	−21.84	−25.99	−17.68	< 0.01
IA	−21.80	−26.09	−17.51	< 0.01
IMA	−16.63	−22.17	−11.09	< 0.01
SCC	−22.16	−22.16	−22.16	< 0.01
Carcinoid	0^a^			

Abbreviations: ENB, electromagnetic navigation bronchoscopy; AIS, adenocarcinoma in situ; CT, computed tomography; IA, invasive adenocarcinoma; IMA, invasive mucinous adenocarcinoma; LLL, left lower lobe; LUL, left upper lobe; mGGO, mixed ground‐grass opacity; MIA, minimally invasive adenocarcinoma; pGGO, pure ground‐grass opacity; RLL, right lower lobe; RML, right middle lobe; RUL, right upper lobe; SCC, squamous cell carcinoma.

^a^ This parameter is set as the reference in subgroup.

### Perioperative results and complications

**FIGURE 3:**
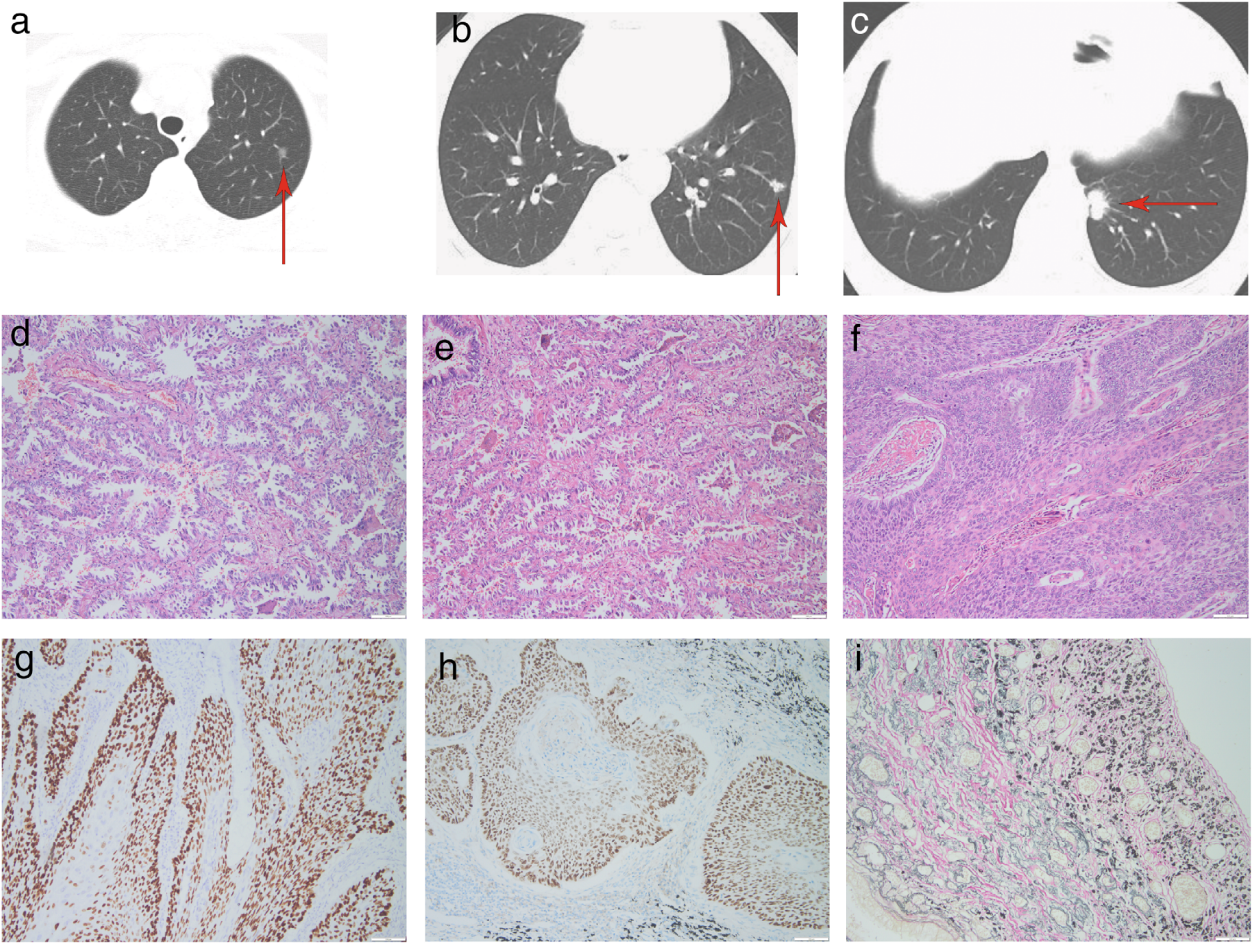
A 74‐year‐old male patient had three unilateral synchronous multiple primary lung cancer. The lesions were in the (a) upper lobe and (b and c) lower lobe of the left lung. The nodules in the upper lobe were removed by electromagnetic navigation bronchoscopy‐guided segmentectomy. (d) The pathology was microinvasive adenocarcinoma. Wedge resection was performed on another nodule in the lower lobe. (e) The pathology of this nodule was invasive adenocarcinoma with papillary pattern. The main lesions were removed by extended segmentectomy. (f) The pathology was moderately differentiated squamous cell carcinoma. Immunohistochemistry results of this tumor was as follows: (g) P40(+); (h) P63(+); (i) elastic fiber staining (+). Magnification: ×10.

After dye labeling guided by ENB, all patients underwent unilateral video‐assisted thoracoscopic sublobectomy. Sublobectomy includes many surgical procedures. To simplify the classification, sublobectomy was divided into wedge resection and segmentectomy. Segmentectomy includes anatomic segmentectomy, nonanatomic segmentectomy, extended segmentectomy, single or combined segmentectomy. All 353 pulmonary nodules in 138 patients were successfully resected. Figure 3 shows the treatment process and pathological feature of patients with SMPLC. There were also 116 (32.86%) cases of adenocarcinoma in situ, 131 (37.11%) minimally invasive adenocarcinoma, and 96 (27.20%) invasive adenocarcinoma, which included lepidic, acinar, papillary, micropapillary, and solid adenocarcinoma were 30, 43, 14, four, and five, respectively. Furthermore, there were some rare cancer types of small lung nodules, including invasive mucinous adenocarcinoma (*n* = 5), followed by squamous cell carcinoma (*n* = 2), carcinoid (*n* = 2), and lung small cell carcinoma (*n* = 1).

We further analyzed the differences in surgical outcomes among patients with different numbers of pulmonary nodules (Table 5). The operation time had a statistical difference as the number of targets increased (*p* = 0.009), 105 (range 75–133), 111 (range 85–148), 95 (range 70–124), 145 (range 135–170) min, respectively. There was no difference in drainage volume on the first postoperative day, with 210 (range 140–280), 235 (range 180–315), 305 (range 180–450), and 200 (range 100–300) mm of two, three, four, and five nodules, respectively. However, the drainage volume on the third postoperative day increased with the number of nodules (*p* = 0.004). Furthermore, postoperative catheter time was statistically prolonged with the increased pulmonary nodule numbers (*p* = 0.01), four (range 4–5), four (range 4–5), five (range 4–5), 7 (range 6–8) days, respectively. Wedge resection was performed on three patients. Moreover, segmentectomy was performed on 70 patients, and 65 underwent combined wedge resection and segmentectomy. There was no difference in hospital stay with various pulmonary nodule numbers, nine (range 8–10), nine (range 8–11), nine (range 8–10), and nine (range 7–10) days, respectively. Postoperative complications occurred in 11 patients, including persistent air leakage (n = 6), postoperative fever (*n* = 4), hemoptysis (*n* = 1), and persistent cough (*n* = 1).

**TABLE 5:** Perioperative outcomes of patients with different numbers of nodules

Outcome	Nodule numbers				*p*‐value
	2	3	4	5	
Operation time	105 (75–133)	111 (85–148)	95 (70–124)	145 (135–170)	0.009
MDV of postoperative 1 days	210 (140–280)	235 (180–315)	305 (180–450)	200 (100–300)	0.09
MDV of postoperative 2 days	150 (100–200)	175 (100–228)	220 (180–280)	200 (170–290)	0.06
MDV of postoperative 3 days	100 (45–145)	100 (73–165)	180 (120–200)	180 (160–220)	0.004
postoperative catheter time (per‐nodule basis)	4 (4–5)	4 (4–5)	5 (4–5)	7 (6–8)	0.01
Hospital stay (per‐nodule basis)	9 (8–10)	9 (8–11)	9 (8–10)	9 (7–10)	0.95
Surgery					
Wedge resection	3	0	0	0	
Segmentectomy	46	17	3	4	
S + W	36	19	7	3	
Complications					
Persistent air leakage	0	1	2	3	
Hemoptysis	0	0	0	1	
Persistent cough	0	0	0	1	
Postoperative fever	1	0	2	1	

Abbreviations: IQR, interquartile range; MDV, Mean drainage volume; S + W, wedge resection + segmentectomy.

## DISCUSSION

With the advancement of high‐resolution CT scanning technologies and the popularization of malignancy screening programs, more and more people are diagnosed with mGGO.^15^, ^16^ According to the International Association for the Study of Lung Cancer, small and multiple ground‐glass opacities found for the first time could be considered SMPLC rather than intrapulmonary metastases.^17^ There are two categories of MPLC, which are classified as synchronous MPLC and metachronous MPLC based on the occurrence time of the lesion.^18^ The reported rate of SMPLC ranges from 0.2% to 8%.^19^, ^20^ After comprehensive therapy, a second primary lung cancer known as metachromatic MPLC might develop in some patients. The overall rate of metachromatic MPLC after lung cancer treatment has previously been reported to be approximately 4%–10%.^21^, ^22^ However, there are no criteria for diagnosis and surgical resection of SMPLC to date. Precise localization and appropriate surgical procedures based on the number and location of nodules that completely resect and preserve lung function are required. Therefore, our center preliminarily explored the efficacy and accuracy of ENB‐guided Uni‐VATS sublobectomy for stage IA SMPLC.

This study included 138 patients with 353 small nodules who underwent ENB guided single‐port thoracoscopic sublobectomy. Electromagnetic navigation localization was performed on 162 nodules of 138 patients. Furthermore, 161 (99.83%) nodules were well stained and successfully localized and 131 were identified as accurately localized. The success rate was similar to that in the Marino et al. study which enrolled 72 nodules measuring ≤2 cm. Compared to traditional CT‐guided puncture localization, ENB can enter the quaternary bronchioles, which is more accurate and precise.^23^, ^24^ To further investigate the effectiveness of ENB in the localization of SMPLC, we made a localization accuracy rating scale. In addition, we attempted to analyze the factors affecting localization accuracy using ordinal logistic regression analysis. Therefore, we analyzed the risk factors of localization including nodule size, distance from nodules to visceral pleura, nodule location, bronchial signs, and postoperative pathological features.

The ordinal regression analysis showed that nodule size and position had no effect on navigation accuracy. This is inconsistent with the study by Yoshiyasu et al. which analyzed 257 nodules in 219 patients and found that the position of the upper lobe of both lungs was a risk factor for navigational markers (*p* < 0.05).^25^ This difference may be related to the operation mode and proficiency of the surgeon. And the risk of localization failure due to nodules location can be gradually reduced by extending the learning curve and improving techniques.^26^, ^27^ Furthermore, we found that the accuracy of nodule localization significantly decreased when there were no bronchus signs (estimate = −1.17, *p* = 0.02). Zhang et al. also performed three‐dimensional image reconstruction of 181 nodules and found that the bronchus signs around nodules could improve the accuracy of electromagnetic navigation and positioning.^28^ Simultaneously, the distance of the nodule from the pleura can affect navigation accuracy (estimate = −1.27, *p* < 0.01). It is challenging to identify subsolid or deep nodules during thoracoscopic surgery.^29^ ENB‐guided dye labeling provides visual markers for thoracoscopic surgery.^30^ The advantages of this approach are painless, simplified workflow, high accuracy, fewer complications, and surgical resection of nodules can be performed in the multifunctional operating room, thus, improving efficiency and safety.^31^, ^32^

SMPLC is a type of malignancy with special pathogenesis and biological behavior. Gao et al. reported that the 5‐year overall survival of patients with SMPLC was 45%, which was completely different from the overall survival of multiple pulmonary nodules (93%).^33^ Currently, the accepted treatment strategy is surgical resection for SMPLC^34^; however, an accurately optimal surgical strategy for SMPLC is still controversial. The American College of Chest Physicians recommended resecting all malignant lesions; however, the extent of surgical resection was not clarified.^12^ Although lobectomy is the standard surgical procedure for primary lung cancer, it remains inconclusive whether it should be performed for secondary tumors, particularly those located in different lobes or lungs, because of concerns regarding impaired lung function.

To the best of our knowledge, this is the first study to report preoperative ENB‐guided localization of pulmonary nodules for sublobectomy. Sublobectomy performed at our center primarily included wedge resection, single segmentectomy, segmentectomy combined with adjacent subsegmentectomy, single and combined subsegmentectomy. Wedge resection was performed on three patients. Segmentectomy was performed on 70 patients, and 65 underwent combined wedge resection and segmentectomy. All 393 pulmonary nodules in 138 patients were successfully resected after ENB localization. We further investigated the effect of the different number of nodules on the short‐term outcome postoperatively. As presented in Table 5, the drainage volume on the third postoperative day increased with the number of nodules (*p* = 0.004). Postoperative catheter time was statistically prolonged with the increased pulmonary nodule numbers (*p* = 0.01), four (range 4–5), four (range 4–5), five (range 4–5), and seven (range 6–8) days.

Meanwhile, we found that ENB‐guided completely thoracoscopic sublobectomy for SMPLC has the following advantages. First, under the guidance of ENB, intraoperative methylene blue staining visualized some invisible and inaccessible nodules, allowing for safer and more accurate resection. Second, this combined operation method reduced the operation time and the patient recovers faster, which was consistent with the concept of ERAS. Third, ENB was a one‐stop positioning and treatment platform, where a single anesthesia can locate and treat the lesion, reducing the patient's radiation exposure and shortening the overall treatment time.

By analyzing the characteristics of postoperative complications, we found that with the increase in nodule numbers, the lung tissue removed by surgery also increased. In addition, 6/7 patients with five pulmonary nodules had varying complications after the total nodule resection, including persistent air leakage, hemoptysis, persistent cough, and postoperative fever. Simultaneously, it also caused us to wonder whether all patients with MPLC undergoing total surgical resection can acquire benefit. Is surgical resection combined with microwave ablation or stereoscopic radiotherapy a more beneficial treatment for SMPLC?

This study had certain limitations. First, it was retrospective with potential selection bias. In addition, our research did not compare the outcomes of other localization techniques combined with sublobectomy to treat SMPLC, including CT or other navigation techniques.

In conclusion, our study showed that ENB‐guided Uni‐VATS sublobectomy is safe and effective when applied to patients with stage IA SMPLC. It provides a new option for accurate preoperative localization of multiple pulmonary nodules. In addition, this technique preserves more lung function while treating multiple lesions and reduces the risk of resection failure. Simultaneously, complete resection of multilocation nodules (such as more than four lesions) increases the risk of postoperative complications. Therefore, a new combined treatment strategy for SMPLC should be explored to ensure the treatment effect and reduce physical trauma.

## CONFLICT OF INTEREST

All authors declare no conflicts of interest in association with the present study.
